# Molecular exploration of host-pathogen interactions in severe *Pseudomonas aeruginosa* infection through a multi-level data integration approach

**DOI:** 10.3389/fmed.2025.1600509

**Published:** 2025-10-14

**Authors:** Francesco Messina, Claudia Rotondo, Luiz Ladeira, Sara Crosetti, Michele Properzi, Valentina Dimartino, Benedetta Riccitelli, Bernard Staumont, Giovanni Chillemi, Liesbet Geris, Maria Grazia Bocci, Carla Fontana

**Affiliations:** ^1^Laboratory of Microbiology and Biobank, National Institute for Infectious Diseases “Lazzaro Spallanzani” IRCCS, Rome, Italy; ^2^Biomechanics Research Unit, GIGA Institute, University of Liège, Liège, Belgium; ^3^Bioinformatics Research Unit in Infectious Diseases, National Institute for Infectious Diseases “Lazzaro Spallanzani” IRCCS, Rome, Italy; ^4^Department of Experimental Medicine, University of Rome “Tor Vergata”, Rome, Italy; ^5^Skeletal Biology and Engineering Research Center, KU Leuven, Leuven, Belgium; ^6^Biomechanics Section, Department of Mechanical Engineering, KU Leuven, Leuven, Belgium; ^7^Intensive Care Unit, National Institute for Infectious Diseases “Lazzaro Spallanzani” IRCCS, Rome, Italy

**Keywords:** *P. aeruginosa*, host-pathogen interaction, bacterial infection, disease map, sepsis

## Abstract

**Introduction:**

Understanding host-pathogen interactions is crucial for explaining the variability in sepsis outcomes, with *Pseudomonas aeruginosa* (*PA*) remaining a significant public health concern. In this work, we explored *PA*-human host interaction mechanisms through a data integration workflow, focusing on protein-protein and metabolite-protein interactions, along with pathway modulation in affected organs during severe infections.

**Methods:**

A scoping literature review enabled us to construct a domain-based infection network encompassing pathogenesis concepts, molecular interactions, and host response signatures, providing a wide view of the relevant mechanisms involved in severe bacterial infections.

**Results:**

Our analysis yielded a literature-based comprehensive description of *PA* infection mechanisms and an annotated dataset of 189 *PA*-human interactions involving 151 proteins/molecules (109 human proteins, 3 human metabolites, 34 *PA* proteins, and 5 *PA* molecules). This dataset was complemented with gene expression analysis from *in vivo PA*-infected lung samples. The results indicated a notable overexpression of proinflammatory pathways and *PA*-mediated modulation of host lung responses.

**Discussion:**

Our comprehensive molecular network of *PA* infection represents a valuable tool for the understanding of severe bacterial infections and offers potential applications in predicting clinical phenotypes. Through this approach combining omics data, clinical information, and pathogen characteristics, we have provided a foundation for future research in host-pathogen interactions and the mechanistic grounds to build dynamic computational models for clinical phenotype predictions.

## Introduction

Sepsis caused by multi-drug resistant pathogens remains a leading cause of mortality in intensive care units (ICU) and represents a significant public health concern (1, 2). While it is established that microbial infection outcomes depend heavily on host conditions and spatial interactions between microbes, hosts, and other microorganisms (3, 4), many molecular details of these complex relationships remain unexplored. *Pseudomonas aeruginosa* (*PA*) is one of the most common pathogens for nosocomial infections, and, along with *Acinetobacter baumannii* and *Enterobacterales* resistant to carbapenems, it was listed among critical priority pathogens for World Health Organization (5, 6). The European Centre for Disease Prevention and Control (ECDC) has included *PA* in its antimicrobial resistance surveillance program (7). As an opportunistic human pathogen particularly affecting Cystic Fibrosis (CF) patients, *PA*’s clinical significance stems from multiple drug resistance mechanisms, numerous virulence factors, and biofilm production capabilities, enhancing its infection and host colonization potential (8). Recently, computational approaches have aided in unraveling mechanistic insights of *PA* infections. A network-assisted experiment allowed the identification of novel genes for virulence and antibiotic resistance, confirmed through experimental validation, showing cross-resistance against multiple drugs due to the same genes (9). In another effort, a real-time deep-learning model was applied to sepsis patients aiming to estimate prognostic outcomes from early infection phases (10). The model addressed baseline acuity, comorbidities, seasonal effects, and secular trends over time, unraveling the strategic significance of computational modeling to improve the clinical outcomes in sepsis patients.

Mechanistic computational modeling, omics data analysis, and clinical research have emerged as crucial tools for bridging the gap between conceptual models and clinical practice in infectious diseases (11, 12). By structuring key pathophysiological mechanisms and identifying conceptual domains, molecular diagrams provide novel insights into biomedical knowledge (11, 13, 14). The value of network-based exploratory and molecular virus-host interactome approaches was particularly evident during the COVID-19 pandemic, where rapid identification of molecular interactions between SARS-CoV-2 and human hosts became crucial to explain the clinical manifestations (15–19), as well as enabled a timely drug repurposing (20, 21). In this context, the resulting molecular maps of disease mechanisms (e.g., a Disease Map)^1^ provided biological meaning to apparently unrelated interactions, facilitating the mechanistic understanding of complex disease processes (22, 23). Following this paradigm, we applied similar strategies to bacterial pathogens such as *PA*, to uncover actionable insights about complex host interactions in severe systemic infections.

Our study presents a data integration workflow to build a molecular map of interaction between *PA* and human hosts in severe infection. Through extensive literature review, data curation, and gene expression meta-analysis, we have documented *PA* infection pathogenic mechanisms, direct protein-protein interactions (PPI), metabolite-protein interactions (MPI), and pathway activations in affected organs, organizing these findings into three conceptual domains: “cellular interaction level”, “tissue interaction level”, and “organ interaction level”.

## Materials and methods

### Scoping review

We conducted independent literature reviews compliant with international reference guidelines for scoping reviews (24). For each domain, the scoping review outcomes were processed to identify features of *PA* interactions with the host and the direct or indirect effects that they cause within the host itself.

Using a structured search string in PubMed (Supplementary Text 1), we identified 532 articles after excluding duplicates, non-English publications, and studies not addressing systemic infection or host-pathogen interactions. We supplemented this with 27 additional articles focusing on host response to *PA* infection in both mouse models and human patients through omics data analysis. During the review process, papers were evaluated in three sequential inclusion criteria: (i) title relevance; (ii) abstract consisting of three conceptual domains, and (iii) identification of specific pathogenic mechanisms in *PA* infection through full-text analysis. The final selection comprised 150 articles which were categorized into three interaction levels: (1) “cell interaction level”; (2) “tissue interaction level”; and (3) “organ interaction level”. Full-text articles were evaluated by the curators to define the best possible conceptual domains, following the reference methodology (PRISMA-ScR) for the assessment (25, 26). Each article selected for review was independently read and evaluated by two reviewers. At the end of the evaluation, the data results were discussed and evaluated in a specific meeting of the entire working group. Each article was assigned a unique reference ID (SR) and documented in Supplementary Table 1.

### Conceptual domains

**FIGURE 1:**
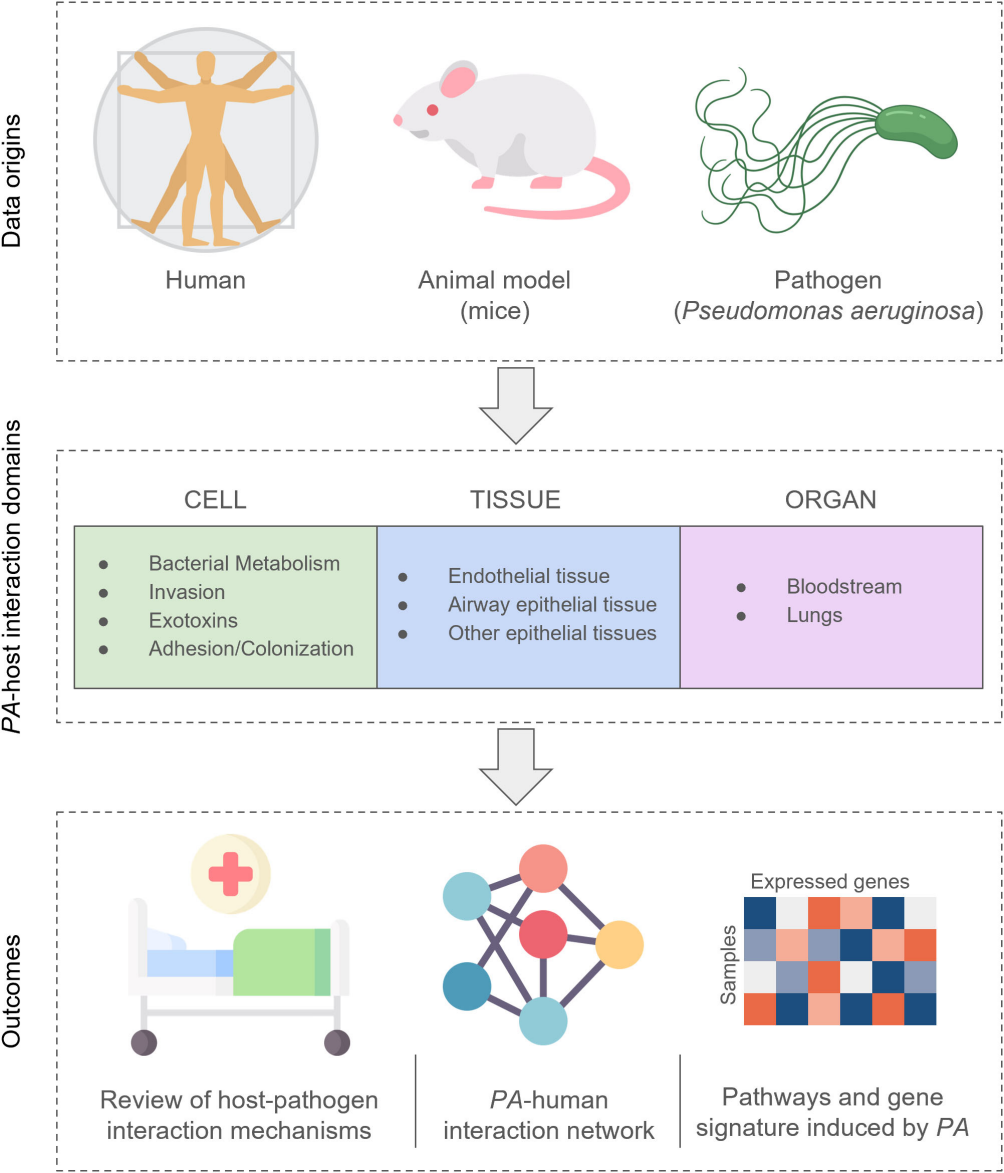
Structure of data collection and analysis workflow. For each level, we identified further conceptual domains, describing the interactions with *PA* molecules.

First, we identified the conceptual domains that organize the information obtained from the literature, providing a hierarchical model of host-pathogen interaction, following a previous experience on mapping host-pathogen interactions in the COVID-19 Disease Map project (11, 12). Three interaction levels within the host’s system were identified: cell, tissue, and organ. For each level, we further identified conceptual domains, describing the interactions with the pathogen (Figure 1). A comprehensive description of all mechanisms and *PA*-human interactions, along with search string, containing all search terms used in the scoping review section on PubMed, and protein abbreviation were reported in Supplementary Text 1, while a summary can be found below in the results section.

### Molecular interaction dataset and human host - *PA* interactome

We documented PPI and MPI between *PA* and humans. All interaction details, including type, Uniprot ID, literature reference, and subdomains of the model, were compiled in the curated dataset (Supplementary Table 2). We constructed a network-based interaction model by exploring *PA*-host data gathered from the scoping review, following methodology established for SARS-CoV-2-human host interactions (16, 17). Human PPI data was retrieved using R packages PSICQUIC and biomaRt (27, 28), resulting in a comprehensive large network of 13,334 nodes and 73,584 interactions that included *PA*-human host interactions. The mechanisms of infection were estimated using the Random Walk with Restart (RWR) algorithm (29), using each *PA* protein as a seed and limiting the output to the 200 closest host proteins per *PA* protein. Network visualizations were generated using GEPHI 0.9.2 (30). Gene set enrichment analysis (GSEA) was performed using the R package enrichR (31), testing against Reactome 2022, KEGG 2021 and WikiPathways 2023 human pathways databases (32–34).

### Meta-analysis of the whole transcriptome from animal model of *PA*-induced sepsis

We performed a meta-analysis of gene expression in mouse lung samples comparing *PA*-infected tissues with healthy controls using data from two projects. The first dataset comprised 12 bulk RNAseq samples from *PA*-infected lung tissues (PRJNA975462; GEO: GSE233206, SRA Study SRP439193) (35), while the second included 6 bulk gene expression samples from acute and chronic *PA* pulmonary infection (PRJNA793679; GEO: GSE192890, SRA Study SRP353174) (36). SRA data was processed using Prefetch and converted to FASTQ files using the fastq-dump tool from the SRA Toolkit software v2.11.0 (37, 38). Reads were aligned to the mm10 mouse reference genome using HISAT2 (39). Differentially expressed genes (DEGs) were identified using DESeq2 v.1.42.1 in R version 3.4.3 (40), with thresholds set at Log2FC > |1| and Benjamin-Hochberg False Discovery Rate < 5% (BH-FDR). To account for batch effects between laboratories, we conducted a meta-analysis using metaRNASeq R packages, combining p-values from the two independent RNA-seq experiments using Fisher methods (41). The analysis focused on 21,010 genes shared between datasets, generating combined BH-adjusted *p*-values and average Log2FC values. Genes meeting the thresholds of Log2FC > |1| and BH FDR < 5% were classified as DEGs.

### Gene enrichment on DEGs in *PA* infection and healthy conditions

To deliver biological meaning from the data, we performed a gene enrichment analysis using Reactome, KEGG, and WikiPathways (32–34). The enrichR R package was used to conduct gene set enrichment analysis, with significance assessed through Fisher exact test (*p*-value) and false discovery rate (*q*-value: adjusted p-value for FDR) (31).

## Results

### Domain-based analysis of *PA*-human host interactions reveals detailed pathogenic mechanisms

To understand in detail *PA* infection pathogenic mechanisms, we reported many *PA*-human host interactions mechanisms, organizing them into three conceptual domains: cellular, tissue, and organ-level interactions.

At the cellular level, four key aspects characterize *PA*-host interaction: (i) bacterial adhesion/colonization (*PA*-Ad); (ii) bacterial invasion and innate immune response of the host (*PA*-In); (iii) *PA* exotoxins activity in infection (*PA*-Ex); (iv) bacterial metabolic mechanisms (*PA*-Met). The pathogenic mechanisms in *PA* infection were assigned to each domain (Table 1). *PA* initiates infection through flagellum and type IV pili adherence, interacting with MUC1 ectodomains via NEU1 modulation (42, 43). The bacterium employs multiple adhesion strategies, including biofilm formation, psl adhesins (44, 45), and various receptors binding to extracellular matrix components (46, 47). During invasion, *PA* modifies host cell membranes through PI3K/PIP3/Akt pathway activation and uses specialized proteins like pilY1 for binding (48). The bacterium’s survival in macrophages relies on mgtC and oprF (49). The exotoxin family (exoS, exoT, exoU, exoY, exoA) facilitates pathogenesis through various mechanisms, including protein ribosylation, cytoskeleton modification, and membrane disruption (50–53).

**TABLE 1:** The table summarizes the main pathogenic mechanisms in *PA* infection for each domain, with comprehensive conceptual analysis provided in the Supplementary Text 1: **(A)** cell interaction level; **(B)** tissue interaction level; **(C)** organ interaction level. This structured approach enabled us to characterize specific mechanisms and experimental models of *PA* infections.

Domain	Subdomain	Key molecules	Biological outcome	References
Cell interaction level	*PA* Adhesion/colonization	Flagellum, pilA	Adherence in upper respiratory tract, interaction with IRF-1	(42, 43, 71)
		Flagellum, NEU1	Modulation of binding between flagellum and MUC1	(43, 71)
		Pilus	Interaction with asialo-GM1, asialo-GM2, glycosphingolipids; MMP7 expression induction	(58, 72–74)
		Psl	Biofilm formation, cell adhesion, flagellin-mediated NF-κB activation	(45)
		estA, oprD, oprG, oprQ, PA3923, Paf	Binding to LAMA1 (α4, α5) and FN1	(46, 47)
		lecA	Binding to Gb3 and GPI- anchored CD59	(75)
		CD18, N-glycans	*PA* uptake facilitation via integrin-mediated uptake	(76)
	*PA* Invasion	mgtC, oprF	Macrophage survival	(49)
		Flagellin	EGFR/TGF-α release, MUC1 phosphorylation, TLR5 association	(77)
		IMPa	Leukocyte rolling adhesion via CD43, CD44, CD55, PSGL-1	(78)
		pumA	NF-κB inhibition, interaction with TIRAP, MyD88, UBAP1	(79)
		LPS	SP-A interaction, TNF-α release limitation	(80)
		LL-37	IL-8 production inhibition, mucA mutagenesis	(81, 82)
		lasB	Protein degradation (elastin, collagen, laminin, IgG, C3, α1-AT, IFN-γ, IL-2)	(83)
		LPS	MUC5AC overproduction	(84)
		CD95/CD95 ligand	Apoptosis triggering, NF-κB/JNKs/GADD153/PLA 2 stimulation	(85)
		PTEN-CFTR complex	*PA* intracellular killing promotion	(86)
	Exotoxins	Azurin	Cell proliferation inhibition via aldolase A secretion	(87)
		3OC12-HSL	T-lymphocyte proliferation inhibition, MAPK-p38 activation	(88)
		PAI-1	Cyclooxygenase 2 activation in fibroblasts and ECs	(89)
		PNC	Neutrophil death, mitochondrial dysfunction, IL-8 downregulation	(51, 90, 91)
		pvrA	PC and fatty acid catabolism regulation	(92)
Tissue interaction level	Endothelial tissue	APOE3	NF-κB reduction in monocytes, antibacterial activity	(54)
		exoS, exoT	Lim kinase-cofilin pathway modulation, GTPase inactivation	(55)
		lasB/pseudolysin	Endothelial adherence disruption, cytotoxicity	(67)
	Airway epithelial tissue	pilY1	PI3K/PIP3/Akt pathway activation, membrane remodeling	(48)
		Flagella	TLR5 activation, neutrophil respiratory burst	(59, 93)
		pilA	Tight junction disruption, IRF-1 activation	(67, 94–96)
		exoA	ADAM10 interaction, leukocyte migration alteration	(97)
		PNC	Ciliary dysfunction, mucus velocity alteration	(98)
		PA-IL, PA-IIL	Cilia binding, airway infection facilitation	(99)
		lasB	EC detachment via FN1/vWf degradation	(47)
		Vav3	b1 integrin/FN1 complex formation in CF	(100)
		CFTR	*PA* uptake regulation, NF- κB activation	(60, 61)
		Various	IL-6/CXCL8/TACE expression induction	(101)
	Other epithelial tissues	HSPGs	Enhanced apical surface binding	(62)
		pilA, Flagella	N-glycan and HSPG- mediated binding	(62)
		T3SS, LPS	Barrier function disruption	(63)
		exoS	Na/K-ATPase inhibition via FXYD3	(102)
		T3SS components	Keratitis development, tight junction disruption	(96)
		Fur regulator	Iron acquisition pathway regulation	(103)
Organ interaction level	Lungs	LPS, CFTR	*PA* uptake, NF-κB activation	(59, 104), (64)
		CFTR/TLR4/TL R5	Phagocytosis regulation, inflammatory response	(59, 60)
		TRPV4	Immune defense enhancement	(65)
		Elastase	IgG cleavage, phagocytosis inhibition	(105)
		LL-37, CLEC5A	NET formation, cytokine release	(66)
		MIF	Lung inflammation reduction	(72)
		Various	Altered immune cell composition, pathway regulation	(35, 36),
		–	Gut microbiota metabolism disruption	(106)
	Bloodstream	TREM-1	Inflammatory response modulation	(69)
		QS genes, pqsH	Systemic infection adaptation	(107)
		Hxu system	BSI pathogenesis regulation	(70)
		–	Blood metabolome alteration	(108)
		–	Differential immune cell response	(68)

At the tissue level, *PA* affects three primary domains: (i) endothelial tissue (Endothelial Tissue - EnT); (ii) lower airway and alveolar epithelial tissue in the lung, including CF conditions (Airway Epithelial Tissue - AET); and (iii) other epithelial tissues such as desquamated bronchial and urinary epithelia (Other Epithelial Tissues - ETs). In endothelial tissue, particularly during severe infection, APOE exhibits antibacterial activity (54), while T3SS affects actin cytoskeleton dynamics (55). The bacterium adapts to blood survival by regulating metabolic pathways and virulence factors (56, 57). In airway epithelial tissue, particularly relevant in CF conditions, *PA* flagella binds to asialoGM1 and MUC1, triggering inflammatory responses (43, 58, 59). CFTR plays a crucial role in *PA* uptake and inflammation (60, 61). In other epithelial tissues, *PA* binds through HSPGs and N-glycans (62), with quorum sensing molecules affecting barrier integrity (63).

Finally, at the organ level, *PA* infection primarily impacts the lung and bloodstream. In lung infections, particularly in CF, *PA* causes intense inflammation with neutrophil infiltration and cytokine production, inducing changes in immune cell composition (36, 59, 64). The infection involves various immune mechanisms, including TRPV4 (65), TIM3/Gal-9 signaling (64), and NET formation (66). In bloodstream infections, *PA* induces differential immune cell responses and affects the vascular endothelium through multiple mechanisms, such as TREM-1 (67–69). The Hxu system contributes significantly to bloodstream infection capability (70). These multi-level interactions highlight the complexity of *PA* pathogenesis and its adaptive capabilities in different host environments.

### *PA*-host proteins interaction network reveals key mechanisms modulated in humans by *PA* severe infection

To reveal key molecular mechanisms in *PA* severe infection, we collected the molecular interactions between *PA* and human proteins during different infection stages, which were manually curated. Analysis of 92 articles revealed multiple direct protein-protein interactions (PPI) and molecule-protein interactions (MPI), detailed in Supplementary Table 2 and annotated with Uniprot IDs, references, and model subdomains.

We identified 151 molecules: 109 human proteins, 3 human metabolites (Gangliotetraosylceramide, Phospholipid cell membrane, glycosphingolipid globotriaosylceramide), 34 *PA* proteins, and 5 *PA* molecules (3O-C12-HSL, LipidA, LPS, Exopolysaccharide, Pyocyanin), yielding 189 *PA*-human interactions and 7 human-human interactions. Note that the 189 interactions include multiple events involving the same molecules, while the 151 components represent unique entities within the network.

These interactions were categorized into four cellular domains: Adhesion process (*PA*-Ad), invasion and injury of tissue (*PA*-Inv), exotoxin production (*PA*-Ex) and bacterial metabolism (*PA*-Meta).

Gene enrichment analysis revealed significant pathway associations across Reactome, WikiPathways and KEGG (Supplementary Table 3). Notable enrichments included the “Pathogenic *Escherichia coli* Infection WP2272” pathway (WikiPathways) and “Pertussis” (KEGG) with FDR < 0.0001%. Reactome analysis highlighted three significant pathways (FDR < 0.0001%), including Programmed Cell Death R-HSA- 5357801, Toll-like Receptor Cascades R-HSA-168898, and Signaling by Interleukins R-HSA-449147. In these pathways several key proteins (e.g., exoS and exoT) would play a modulating role, such as inhibition of interleukin proteins or degradation of occludin (OCLN), a cell death regulator (109).

**FIGURE 2:**
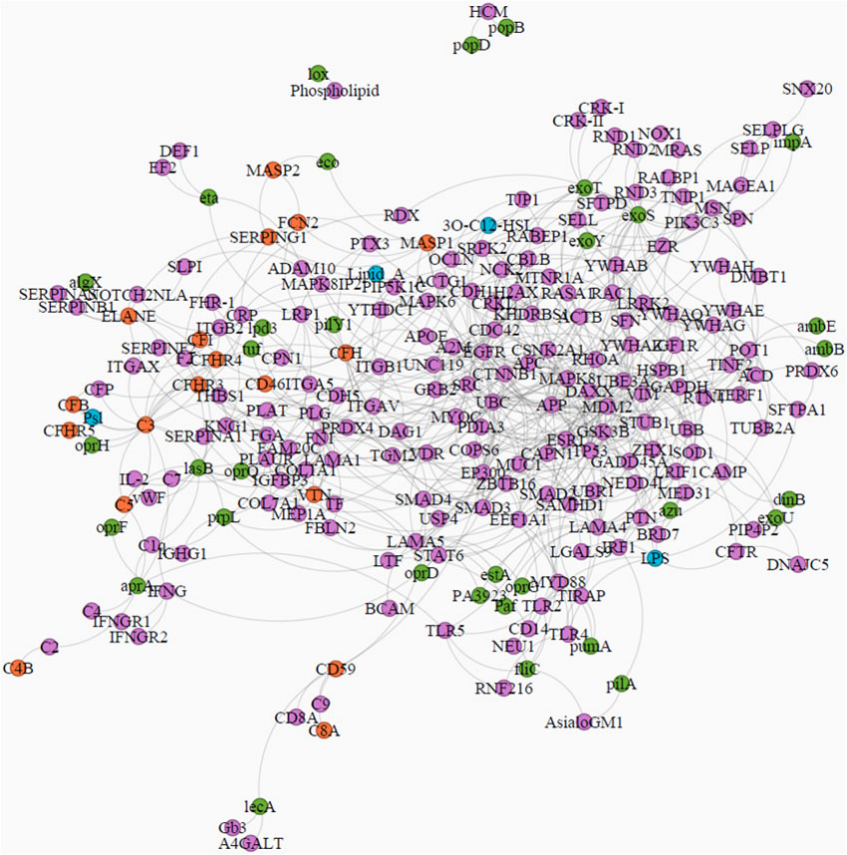
Network of *PA*-human host molecular interactions, with the top 200 nearest proteins found by the Random Walk with Restart (RWR) algorithm. Nodes have different colors to show different kinds of molecules: purple, human proteins; green, *PA* proteins; light blue, *PA* molecules; orange, human proteins belonging to the complement pathway.

A full network of interactions between *PA* and human host proteins (Figure 2) enabled us to reveal the overall cell response to infection, digging up also new possible pathogenic mechanisms: the modulating effect of outer membrane proteins oprH, oprQ, and the elastase lasB on Complement Cascade Pathway (Reactome R-HSA-166658; 18/55; FDR < 0.0001%) for contrasting bacterial cell damage. These proteins also showed significant interactions with blood clotting factors, such as VWF, SERPINF2, PLAUR, PLAT, and PLG (Complement and Coagulation Cascade WP558; 20/58; FDR < 0.0001%), suggesting a potential involvement in thrombotic event. Furthermore, the role of exotoxin (exoS, exoY, and exoT) in *PA* infection proved central to triggering of cell toxicity through interactions with cytoplasmic 14-3-3 proteins (e.g., YWHAB).

### Meta-analysis of whole transcriptome of *PA*-infected lung tissues from mice reveals selective modulation of pro-inflammatory pathways

**FIGURE 3:**
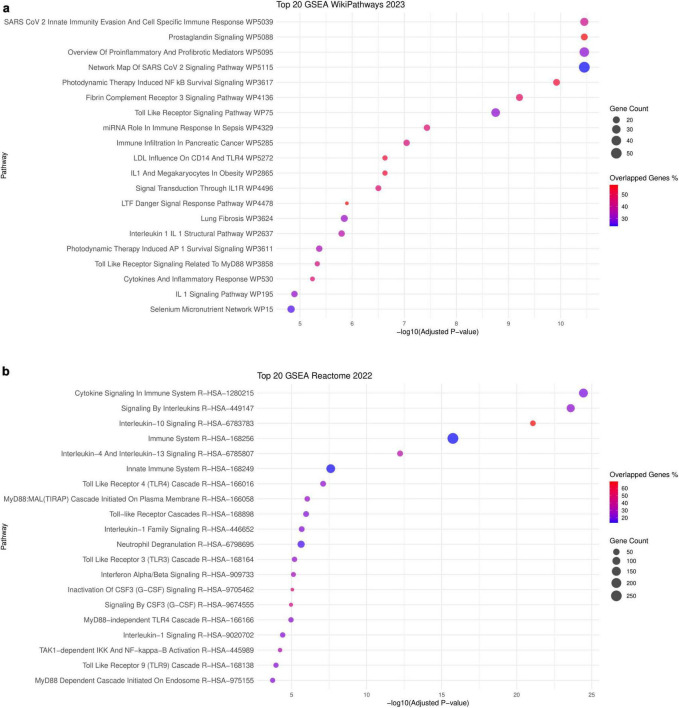
GSEA with WikiPathways **(a)** and Reactome **(b)**, based on upregulated DEGs in *PA* - infected samples, obtained from meta-analysis of two infection experiments in mouse lung tissues.

To better define the biological response in *PA*-infected lung tissues, we carried out a meta-analysis of gene expression of two bulk RNAseq datasets (GSE233206 and GSE192890) comparing *PA*-infected mice lung samples with healthy controls. Our meta-analysis identified 1,560 upregulated and 383 downregulated genes (Log2FC > 1; FDR BH < 5%, Supplementary Table 4). Pathway analysis of upregulated genes using WikiPathways revealed significant enrichment in inflammation-related pathways, notably “Overview of Proinflammatory and Profibrotic Mediators WP5095” (39/129, FDR < 0.0001%). Reactome analysis aligned with our scoping review findings, highlighting significant enrichment (FDR < 0.000001%) in key pathways: Cytokine Signaling in Immune System R-HSA-1280215 (145/702), Signaling by Interleukins R-HSA-449147 (109/453), Interleukin-10 Signaling R-HSA-6783783 (31/45) (Figures 3a, b). Proinflammatory pathways were found nested into Interleukins R-HSA-449147 (*Homo sapiens*) Reactome’s entry (Interleukin-2 family signaling R-HSA-451927; Interleukin-3, Interleukin-5 and GM-CSF signaling R-HSA-512988; Interferon alpha/beta signaling R-HSA-909733; Interferon gamma signaling R-HSA-877300; ISG15 antiviral mechanism (Homo sapiens) R-HSA-1169408; PKR-mediated signaling R-HSA-9833482; TNFR2 non-canonical NF-kB pathway R-HSA-5668541; Signaling by CSF1 (M-CSF) in myeloid cells; R-HSA-9680350. All these pathways have many key proteins for *PA* infection, which are described as targets for *PA* exoU, exoS, azu, lasB, aprA, oprF, pilA, and LPS. These results suggest that these pathways are directly involved in initiating the innate response to *PA* infection, but also highlight the potential role of *PA* molecules in modulating and limiting this response, particularly for interleukin signaling.

## Discussion

In this work, we present the development of a comprehensive data integration model to understand *PA* infection through detailed exploration of the literature and metanalysis of transcriptomics datasets, identifying specific human molecular targets for each *PA* molecule, pathogenic mechanisms, and host responses. In general, *PA* could be considered a useful example for studying severe systemic infections, given its multi-drug resistance capabilities, ability to cause acute and chronic infections in pulmonary disease patients, and its capacity to form biofilm in hypoxic conditions, which makes it extremely difficult to treat (110, 111).

Firstly, the central role of exoS during infection was confirmed, while enhanced activity among exo family proteins, including exoY and exoT, was widely highlighted (112). ExoS functions by inhibiting several proteins of interleukin pathways and inducing the degradation of Occludin (OCLN), an integral membrane protein involved in cytokine-induced regulation of the tight junction permeability barrier, ultimately inducing cell death (67). Through its ADP RT activity, exoS modulates host cell apoptosis, inducing *PA*-infected cell death by targeting various Ras proteins (113). The Complement Cascade Pathway undergoes modulation by *PA*’s outer membrane proteins, oprH, oprQ, and elastase lasB, which trigger cytotoxic effects and adhesion through complement binding, particularly C3 (114). This result mirrors the mechanism of activation of the complement system, in which C3 is the main actor against bacteria, through a link with oprF, a porin involved in ion transport (Na+ and Cl−) and anaerobic biofilm production (115, 116). A significant finding was the interaction between oprH, oprQ, and lasB with coagulation proteins, suggesting their involvement in thrombotic processes. *PA* lasB’s cleavage of a C-terminal peptide FYT21 derived from thrombin inhibits activation of the transcription factors NFκ-B and activator protein 1 (AP-1). *PA* demonstrates sophisticated modulation of host immune responses through multiple pathways; aprA, lasB, and exoS exhibit inhibitory effects on interleukin pathways (112, 117, 118), indicating an adaptive modulation that enhances *PA* survival within the host. Such an effect was confirmed in *PA* infection, where *PA*-derived DnaK negatively regulates IL-1β production by cross-talk between JNK and PI3K/PDK1/FoxO1 pathways (119). Notably, decreased *PA* levels in CF patients correlate with reduced proinflammatory cytokines (120).

Our findings provided a broader view of molecular perturbations in *PA* systemic infection and served as a foundation for developing specific disease maps for severe *PA* infection, supporting the integration of omics data from clinical cases into predictive computational models. Future developments may incorporate text mining and AI-assisted analysis for drug target identification (23) and digital modeling of the human immune system under infection conditions (121) to better predict real patient outcomes and test potential therapeutic strategies in a personalized fashion.

There are some limitations worth noting. While we have documented numerous significant *PA*-human interactions, our model may not encompass all possible interactions. The PPI/MPI dataset requires iterative updates to incorporate new experimental findings from both *in vitro*, *in vivo* and clinical studies. Furthermore, since our interaction data derives primarily from *in vitro* experiments, the described pathogenic mechanisms require validation in the context of severe systemic infections. Finally, our differential expression meta-analysis, conducted in mouse models with limited sample size, provides an overview of host gene-expression signatures in *PA* infection but requires confirmation through clinical data.

In conclusion, our study provides a comprehensive collection and analysis of molecular mechanisms in *P. aeruginosa* infection, combining literature-based evidence, protein-protein interaction analysis, and transcriptomic data from *in vivo* studies. A detailed dataset of *PA*-host interactions across cellular, tissue, and organ levels was built through a systematic data integration approach. Our findings highlight the complex interplay between *PA* virulence factors and host responses, particularly the role of exoS in modulating interleukin pathways and the involvement of outer membrane proteins in the complement cascade. The integration of differential expression analysis from mouse models further strengthens our understanding of host response patterns, particularly in proinflammatory and immune signaling pathways. As antimicrobial resistance continues to pose significant challenges in healthcare, such a comprehensive molecular understanding may prove invaluable for applying precision medicine approaches to severe bacterial infections and improving patient-tailored treatments in severe systemic infections.

## Data Availability

The datasets presented in this study can be found in online repositories. The names of the repository/repositories and accession number(s) can be found in the article/Supplementary material.

## Footnotes

1
https://disease-maps.io/
