# Supplementary material for: Molecular exploration of host-pathogen interactions in severe Pseudomonas aeruginosa infection through a multi-level data integration approach
Source: Front Med (Lausanne). 2025 Oct 14;12:1600509. doi: 10.3389/fmed.2025.1600509 (PMC12558848; doi:10.3389/fmed.2025.1600509)
Supplement: Supplementary file 3 [file Table_3.docx]

### S-Table 3: the top ten most enriched pathways identified using KEGG 2021, WikiPathway 2023, and Reactome 2022 databases (A, B, and C, respectively).

| A) |  |  |  |  |
| --- | --- | --- | --- | --- |
| KEGG 2021 Pathway | Overlap | P. value | Adj. P.value | Genes |
| Pertussis | 16/76 | 3.63E-19 | 7.15E-17 | ITGB1 JUN FOS MAPK14 RHOA TIRAP C2 C3 C5 CASP3 MAPK1 CD14 SFTPA1 TLR4 MYD88 MAPK3 |
| Pathogenic Escherichia coli infection | 21/197 | 2.12E-18 | 2.08E-16 | ITGB1 JUN FOS F2 MAPK14 ACTB RHOA TIRAP TNFRSF1A CDC42 TJP1 OCLN CASP8 CASP3 MAPK1 RAC1 EZR TLR5 TLR4 MYD88 MAPK3 |
| Proteoglycans in cancer | 21/205 | 4.86E-18 | 3.19E-16 | ITGB1 RDX FN1 PLAUR MSN MAPK14 ACTB RHOA COL1A1 CDC42 VTN MRAS CASP3 MAPK1 CTNNB1 RAC1 EZR TP53 TLR4 TLR2 MAPK3 |
| Salmonella infection | 21/249 | 2.65E-16 | 1.31E-14 | JUN RIPK3 FOS MAPK14 ACTB RHOA TIRAP TNFRSF1A CDC42 CASP8 CASP3 MAPK1 CTNNB1 PIK3C3 CD14 RAC1 TLR5 TLR4 MYD88 TLR2 MAPK3 |
| Shigellosis | 19/246 | 3.81E-14 | 1.50E-12 | ITGB1 JUN MAPK14 ACTB RHOA CRKL TNFRSF1A C3 CDC42 UBB MAPK1 PIK3C3 CD14 RAC1 TLR5 TP53 TLR4 MYD88 MAPK3 |
| Lipid and atherosclerosis | 18/215 | 4.62E-14 | 1.52E-12 | JUN FOS MAPK14 RHOA TIRAP TNFRSF1A CDC42 CASP8 CASP3 MAPK1 CD14 RAC1 TP53 TLR4 MYD88 NOX1 TLR2 MAPK3 |
| Viral carcinogenesis | 17/203 | 2.42E-13 | 6.82E-12 | YWHAE JUN YWHAB YWHAZ RHOA C3 CDC42 CASP8 YWHAQ CASP3 MAPKAPK2 MAPK1 RAC1 TP53 YWHAG YWHAH MAPK3 |
| Tuberculosis | 16/180 | 5.04E-13 | 1.24E-11 | MAPK14 RHOA TIRAP TNFRSF1A C3 CASP8 IFNG CASP3 MAPK1 PIK3C3 CD14 TLR4 MYD88 CAMP TLR2 MAPK3 |
| Hepatitis C | 15/157 | 9.68E-13 | 2.05E-11 | YWHAE YWHAB YWHAZ TNFRSF1A OCLN CASP8 YWHAQ IFNG CASP3 MAPK1 CTNNB1 TP53 YWHAG YWHAH MAPK3 |
| Toll-like receptor signaling pathway | 13/104 | 1.0E-12 | 2.05E-11 | JUN FOS MAPK14 TIRAP CASP8 MAPK1 CD14 RAC1 TLR5 TLR4 MYD88 TLR2 MAPK3 |
|  |  |  |  |  |
| B) |  |  |  |  |
| Wikipathway_2023 Pathway | Overlap | P. value | Adj. P.value | Genes |
| AGE RAGE Pathway WP2324 | 14/66 | 5.85E-17 | 2.55E-14 | JUN MSN MAPK14 RHOA TIRAP SOD1 CDC42 CASP8 CASP3 MAPK1 RAC1 EZR MYD88 MAPK3 |
| Pathogenic Escherichia Coli Infection WP2272 | 13/55 | 1.69E-16 | 3.70E-14 | ITGB1 YWHAZ ACTB RHOA CDC42 OCLN YWHAQ CDH1 CTNNB1 CD14 EZR TLR5 TLR4 |
| CKAP4 Signaling Pathway Map WP5322 | 14/109 | 9.19E-14 | 1.34E-11 | ITGB1 JUN MAPK14 TNFRSF1A TJP1 OCLN CASP8 CDH1 CASP3 MAPK1 CTNNB1 CCN2 VIM TP53 |
| Netrin UNC5B Signaling Pathway WP4747 | 19/99 | 1.45E-13 | 1.59E-11 | COL1A1 CDH5 JUN CASP3 MAPK1 RAC1 CCN2 MAPK14 TP53 RHOA MAPK3 |
| Complement System WP2806 | 13/95 | 3.15E-13 | 2.75E-11 | FGA LAMA5 SELPLG CFH PLAUR PLG C2 C3 VTN C5 SFTPA1 ELANE TLR2 |
| Toll Like Receptor Signaling Pathway WP75 | 13/102 | 8.06E-13 | 5.87E-11 | JUN FOS MAPK14 TIRAP CASP8 MAPK1 CD14 RAC1 TLR5 TLR4 MYD88 TLR2 MAPK3 |
| VEGFA VEGFR2 Signaling WP3888 | 22/430 | 1.46E-12 | 9.10E-11 | ITGB1 YWHAE FGA JUN FN1 PLAUR ADAM10 MAPK14 RHOA RND1 CDC42 CDH5 OCLN MAPKAPK2 MAPK1 CTNNB1 CCN2 RAC1 CCN1 EZR ALDOA MAPK3 |
| Focal Adhesion WP306 | 16/199 | 2.38E-12 | 1.30E-10 | ITGB1 LAMA5 JUN VWF LAMA4 FN1 ACTB RHOA CRKL COL1A1 CDC42 VTN MAPK1 CTNNB1 RAC1 MAPK3 |
| Gastrin Signaling Pathway WP4659 | 13/115 | 3.87E-12 | 1.88E-10 | ITGB1 JUN FOS MAPK14 RHOA CDC42 TJP1 CDH1 CASP3 MAPK1 CTNNB1 RAC1 MAPK3 |
| Hepatitis B Infection WP4666 | 14/150 | 7.9E-12 | 3.3238E-10 | JUN YWHAB FOS MAPK14 YWHAZ TIRAP CASP8 YWHAQ CASP3 MAPK1 TLR4 MYD88 TLR2 MAPK3 |
|  |  |  |  |  |
| C) |  |  |  |  |
| Reactome 2022 Pathway | Overlap | P.value | Adj. P.value | Genes |
| Programmed Cell Death R-HSA-5357801 | 19/208 | 1.75E-15 | 1.44E-12 | YWHAE RIPK3 YWHAB YWHAZ TJP1 OCLN CASP8 UBB YWHAQ CASP3 MAPK1 CTNNB1 VIM TP53 TLR4 ELANE YWHAG YWHAH MAPK3 |
| Toll-like Receptor Cascades R-HSA-168898 | 17/162 | 5.67E-15 | 1.81E-12 | FGA JUN RIPK3 FOS MAPK14 TIRAP CASP8 UBB MAPKAPK2 MAPK1 PIK3C3 TLR5 TP53 TLR4 MYD88 TLR2 MAPK3 |
| Signaling By Interleukins R-HSA-449147 | 25/453 | 6.61E-15 | 1.81E-12 | ITGB1 LAMA5 CRKL CDC42 MUC1 CASP8 UBB CASP3 MAPK1 LGALS9 HAVCR2 MAPK3 JUN FN1 MSN FOS MAPK14 YWHAZ TNFRSF1A SOD1 IFNG MAPKAPK2 VIM TP53 MYD88 |
| Apoptosis R-HSA-109581 | 17/178 | 2.75E-14 | 5.67E-12 | YWHAE YWHAB YWHAZ TJP1 OCLN CASP8 UBB YWHAQ CASP3 MAPK1 CTNNB1 VIM TP53 TLR4 YWHAG YWHAH MAPK3 |
| Immune System R-HSA-168256 | 48/1943 | 4.42E-14 | 7.27E-12 | ITGB1 LAMA5 SERPINA1 CFH YWHAB CBLB TREM1 CRKL C3 CDC42 VTN C5 MUC1 CASP8 UBB CASP3 MAPK1 LGALS9 ELANE HAVCR2 MAPK3 FGA JUN RIPK3 FN1 PLAUR ADAM10 MSN FOS MAPK14 YWHAZ MUC5AC RHOA TIRAP TNFRSF1A SOD1 IFNG DNAJC5 MAPKAPK2 CTNNB1 PIK3C3 VIM TLR5 TP53 TLR4 MYD88 LTF TLR2 |
| MyD88:MAL(TIRAP) Cascade Initiated On Plasma Membrane R-HSA-166058 | 14/112 | 1.35E-13 | 1.85E-11 | FGA JUN FOS MAPK14 TIRAP CASP8 UBB MAPKAPK2 MAPK1 TP53 TLR4 MYD88 TLR2 MAPK3 |
| Toll Like Receptor 4 (TLR4) Cascade R-HSA-166016 | 15/140 | 1,77E-13 | 2.09E-11 | FGA JUN RIPK3 FOS MAPK14 TIRAP CASP8 UBB MAPKAPK2 MAPK1 TP53 TLR4 MYD88 TLR2 MAPK3 |
| Intrinsic Pathway For Apoptosis R-HSA-109606 | 11/55 | 2.83E-13 | 2.91E-11 | YWHAE CASP8 YWHAQ YWHAB CASP3 MAPK1 YWHAZ TP53 YWHAG YWHAH MAPK3 |
| Innate Immune System R-HSA-168249 | 33/1035 | 1.30E-12 | 1.19E-10 | SERPINA1 CFH TREM1 C3 CDC42 VTN C5 MUC1 CASP8 UBB MAPK1 ELANE MAPK3 FGA JUN RIPK3 PLAUR ADAM10 FOS MAPK14 MUC5AC RHOA TIRAP DNAJC5 MAPKAPK2 CTNNB1 PIK3C3 TLR5 TP53 TLR4 MYD88 LTF TLR2 |
| Chk1/Chk2(Cds1) Mediated Inactivation of Cyclin B:Cdk1 Complex R-HSA-75035 | 07/13 | 2E-12 | 1.60E-10 | YWHAE YWHAQ YWHAB CDC25C YWHAZ YWHAG YWHAH |
